# Continuous imaging to evaluate growth and drug responses of patient-derived colorectal tumouroids

**DOI:** 10.1016/j.esmogo.2025.100137

**Published:** 2025-03-03

**Authors:** B.C. Sakshaug, E. Folkesson, T.H. Haukaas, M.S. Sigfúsdóttir, H.H. Trøen, S.B. Sperstad, H.C.H. Bwanika, C. Ringers, I.A. Bergstrøm, G. Klinkenberg, T. Visnes, Å. Flobak

**Affiliations:** 1 Department of Clinical and Molecular Medicine, Norwegian University of Science and Technology, Trondheim; 2 Department of Biotechnology and Nanomedicine, SINTEF Industry, Trondheim; 3 The Cancer Clinic, St Olav’s University Hospital, Trondheim, Norway

**Keywords:** colorectal cancer, patient-derived tumouroids, chemotherapy, imaging, personalised medicine

## Abstract

**Background:**

The use of patient-derived tumouroids is expected to have major implications in preclinical drug testing and clinical decision support. Data acquisition from these samples in drug screens, however, is often limited by time-consuming procedures, scarce screening material, and destructive, single-parameter endpoint measurements.

**Design:**

We seek to increase data collected from patient-derived tumouroids and here present a method for non-destructive, continuous image-based analysis of colorectal tumouroid growth and shape under chemotherapeutic perturbations, conducted within a clinically relevant timeframe.

**Results:**

We assessed several readouts automatically derived from continuous imaging data and concluded that tumouroid growth, and the effect of growth inhibiting drugs, can be robustly monitored by measuring the total tumouroid-covered area in images. We also found that measures of average tumouroid size, diameter, and perimeter provide complementary insights.

**Conclusions:**

Our tumouroid analysis method offers a strategy to maximise data extraction from non-destructive imaging techniques while preserving tumouroids for future research, all within a clinically relevant timeframe.

## Highlights

• There is substantial variation in sample growth depending on which reagents are used in processing.
• Growth of unperturbed samples can be quantified using non-destructive image-based readouts.
• Dose-dependent growth of samples upon exposure to SN-38 can be quantified using the same image-based readouts.
• Image-based readouts relating to tumouroid dimensions can be used for fitting dose–response models.

## Introduction

Patient-derived tumouroids (PDTs) developed from patient cancer biopsies have emerged as a promising model for predicting individual treatment response, enabling the personalisation of cancer treatment. By evaluating treatment response *in vitro*, PDTs show potential for prioritising treatment by identifying effective agents and excluding ineffective ones. Our research focuses on colorectal cancer, where over recent years the modelling capacity of PDTs has been validated in several studies^1^, ^2^, ^3^, ^4^, ^5^, ^6^, ^7^, ^8^, ^9^, ^10^, ^11^, ^12^, ^13^, ^14^, ^15^, ^16^, ^17^, ^18^, ^19^, ^20^, ^21^, ^22^ through retrospective patient tumouroid comparisons. Additionally, a few studies have used tumouroid assays to prospectively assign patients to treatment,^8^^,^^10^^,^^12^^,^^14^^,^^22^ with mixed success. Although limited, current evidence underscores the potential of these models to predict patient responses to anticancer treatment. A systematic review from our group on colorectal cancer tumouroids estimated the sensitivity and specificity of the PDT model for modelling patient responses at 0.79 and 0.75, respectively.^23^

Despite the PDT models’ promise, several obstacles must be addressed before they can become part of routine clinical practice. A major hurdle is the lack of standardised cultivation protocols. Although most groups use similar cultivation methods, local adaptations in protocol parameters and study design vary between groups.^23^ Although some groups have done substantial work in protocol development,^24^ inadequate reporting on major and minor protocol adaptations and reagent choices in the field in general complicates cross-group learning.

Additionally, most protocols use destructive endpoint readouts such as CellTiter-Glo (Supplementary Table S1, Supplementary File A: Materials and Methods, available at https://doi.org/10.1016/j.esmogo.2025.100137) to determine drug responses in PDTs. Designing PDT cultivation and drug screening to maximise data collection without compromising tumouroid integrity, however, is crucial, as rescreening is generally not feasible. In contrast to destructive methods, label-free imaging offers a promising approach for non-destructive, continuous data collection, enabling ongoing response assessments and preserving tumouroids for future analysis and experiments. Despite these advantages, these methods are less frequently reported in the literature.^5^^,^^7^^,^^14^^,^^21^^,^^25^

In the present work, we developed a method for continuous and non-destructive monitoring of tumouroid growth with and without exposure to the chemotherapeutic agent SN-38, the active metabolite of irinotecan. Additionally, we provide insights into the decisions made in establishing our cultivation protocol, aiming to set a standard for future tumouroid research.

## Methods

### Patient material

Tumour material from previously untreated patients undergoing surgical resection with curative intent, either total or hemicolectomy, of stage I-IV colorectal cancer at the St. Olav’s University Hospital, Trondheim, Norway, was obtained following patients’ written informed consent. The study was approved (ref. 2019/246, REK ID 10447) by the Regional Committees for Medical and Health Research Ethics (REC). A total of nine samples were included in the present paper.

### Sample processing and cultivation

Tumour samples 1-9 were processed and cultivated as described below, with some variations depending on whether the sample was used for protocol optimisation or drug-response testing. A detailed description of methods, as well as a list of equipment and reagents can be found in Supplementary File A: Materials and Methods, Table S2, available at https://doi.org/10.1016/j.esmogo.2025.100137. Briefly, following resection, the sample was collected in Dulbecco's Modified Eagle Medium (DMEM) supplemented with 100 U/ml penicillin–streptomycin. For samples 4-9, 10 μM of Y-27632 (ROCK-inhibitor) was added as well. The sample was mechanically digested using scalpels and needles, followed by enzymatic digestion with collagenase type II or Liberase DH. Digestion was followed by serial filtration using 500, 200, and 40 μm filters, out of which the 40-200 μm fraction was kept for subsequent steps. After pelleting in a centrifuge (200-800*g*, 4°C, 5 min), the tumouroids were resuspended in a 1 : 1 mixture of proteinaceous component (Matrigel, Geltrex, Cultrex, or Cellmatrix type I-A), and a medium component [serum-free stem cell medium (SFSCM) or DMEM/F-12 HEPES + 1% bovine serum albumin (BSA)] (Supplementary Table S3, Supplementary File A: Materials and Methods, available at https://doi.org/10.1016/j.esmogo.2025.100137). The tumouroid suspension was seeded in 50 μl droplets at a density of ∼500-2000 tumouroids/droplet in 24-well plates (1 droplet per well, with tumouroids counted manually using a cell counting chip). Droplets were overlaid with 500 μl of growth medium; either SFSCM, IntestiCult^TM^ (commercially available organoid growth medium) or an in-house organoid growth medium after recipes from collaborators^26^ (hereafter referred to as ‘Organoid medium’). For tumouroids subjected to drug response screening, the medium was replaced with the same volume of drug-containing medium following 2 days in culture. Drug-containing medium was removed after 7 days and replaced with fresh growth medium. For tumouroids evaluated for continuous growth, the medium was changed every 3-4 days. For preparation of growth medium, we refer to Supplementary File A: Materials and Methods, Tables S4-S6, available at https://doi.org/10.1016/j.esmogo.2025.100137.

Sample processing and cultivation procedures specific to protocol optimisation are outlined in Supplementary File A: Materials and Methods, available at https://doi.org/10.1016/j.esmogo.2025.100137. All samples included in the study are listed in Supplementary Table S7, available at https://doi.org/10.1016/j.esmogo.2025.100137https://doi.org/10.1016/j.esmogo.2025.100137 (Supplementary File A: Materials and Methods, available at https://doi.org/10.1016/j.esmogo.2025.100137).

### Drug perturbation

For drug response screening, the SFSCM was replaced with SFSCM containing SN-38 in a 5-step, 10-fold concentration gradient ranging from 0.1 to 1000 nM SN-38, with 0.5% DMSO. SFSCM containing 0.5% DMSO was used as vehicle control. Each condition was examined in four technical replicates.

### Imaging and image analysis

Imaging for monitoring sample growth was carried out using an ImageXpress Micro Confocal High-Content Imaging System or an EVOS FL Auto 2 Cell Imaging System. Confocal imaging was carried out at 37°C with a 2× objective and Z-stack images of each well were captured automatically. For each plate, a Z-stack interval was defined so that all tumouroids in the gel droplets were included. Following imaging, each stack of images was converted into a 2D projection image using a ‘best focus’ protocol. 2D projection images were exported as TIFF files and analysed in ImageJ.^27^ For further details regarding the image analysis process, we refer to Supplementary File A: Materials and Methods, available at https://doi.org/10.1016/j.esmogo.2025.100137.

### Data analysis

Data analysis was carried out using R version 4.2.1. R packages used for data analysis and graphics are listed in Supplementary Table S8, available at https://doi.org/10.1016/j.esmogo.2025.100137 (Supplementary File A: Materials and Methods, available at https://doi.org/10.1016/j.esmogo.2025.100137). *Relative total area* was used as the main readout for assessment of tumouroid growth and response, which was computed by dividing the total area covered by tumouroids in each well by the area covered by tumouroids in the same well at day 1 of the experiment.

Average tumouroid area, size, diameter, perimeter, and circularity were used as additional readouts for assessment of tumouroid growth and response. Here, ‘average’ refers to the average of each of the parameters (size, diameter, perimeter, and circularity) across all tumouroids per well, or as a whole-well readout (average area). For each well, we report average parameter values as absolute values per day, i.e. not relative to day 1.

In the Results section, all readouts are reported as the average of technical replicates. For generation of dose–response curves, data were normalised to the mean of the technical replicates of the vehicle control. Dose–response curves were fitted with a five-parameter log-logistic function using the DRC-package in R.^28^

## Results

### Overview of the study

**Figure 1:**
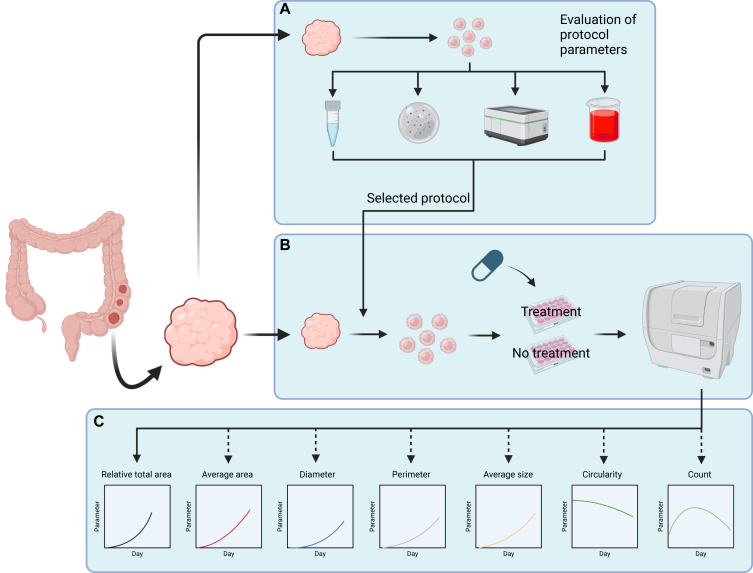
**Overview of the study.** (A) Evaluation of sample processing parameters (digestion enzyme, extracellular matrix, imaging platform, growth medium) using nine PDT samples. Parameters/reagents that promoted the strongest tumouroid growth, or were the simplest to prepare, were selected for remaining samples. (B) Three of the PDT samples were further cultivated in the presence and absence of SN-38 using the selected protocol, and their growth was monitored using confocal microscopy. (C) Growth and response of tumouroids were evaluated using image-based readouts, namely relative total area, average area, diameter, perimeter, size, circularity, and count of the tumouroids. Relative total area (solid arrow) and average area (dashed arrow) represent whole-image readouts, whereas other readouts (dashed arrows) represent average measures across all tumouroids detected per image. Created in BioRender. Sakshaug, C. (2024) https://BioRender.com/m79y621. PDT, patient-derived tumouroid.

The present study evaluated the feasibility of assessing growth and treatment response of PDTs using different image-based readouts and cultivation methods. To ensure that tumouroids exhibited detectable growth, we first evaluated the effect of different processing and cultivation procedures on tumouroid growth (Figure 1A) to select the optimal procedure for future sample processing. Using the selected protocol, we processed and cultivated three independent samples, which were then subjected to growth monitoring via imaging under both perturbed (treated with SN-38) and unperturbed (untreated) conditions (Figure 1B). In total, we assessed tumouroid growth and drug response through seven image-based readouts (Figure 1C).

### Sample processing affects tumouroid growth

To study PDT growth and treatment response through imaging, we began by comparing protocols used by Kondo et al.^29^^,^^30^ and Jeppesen et al.,^25^ as well as other tumouroid and organoid protocols.^1^, ^2^, ^3^, ^4^, ^5^, ^6^, ^7^, ^8^, ^9^, ^10^, ^11^, ^12^, ^13^, ^14^, ^15^, ^16^, ^17^, ^18^, ^19^, ^20^, ^21^, ^22^ We investigated the impact of different digestion enzymes (Liberase DH versus collagenase type II), extracellular matrices (ECM; Cellmatrix Type I-A, Matrigel, Geltrex, and Cultrex), and growth media (IntestiCult, Organoid medium, SFSCM) on tumouroid growth, to determine an optimal procedure for producing tumouroids with detectable growth in image-based analyses. Samples were treated as described in Protocol optimisation—Selection of digestion enzyme, ECM, and growth medium (Supplementary Figure S1, Supplementary File A: Materials and Methods, available at https://doi.org/10.1016/j.esmogo.2025.100137).

**Figure 2:**
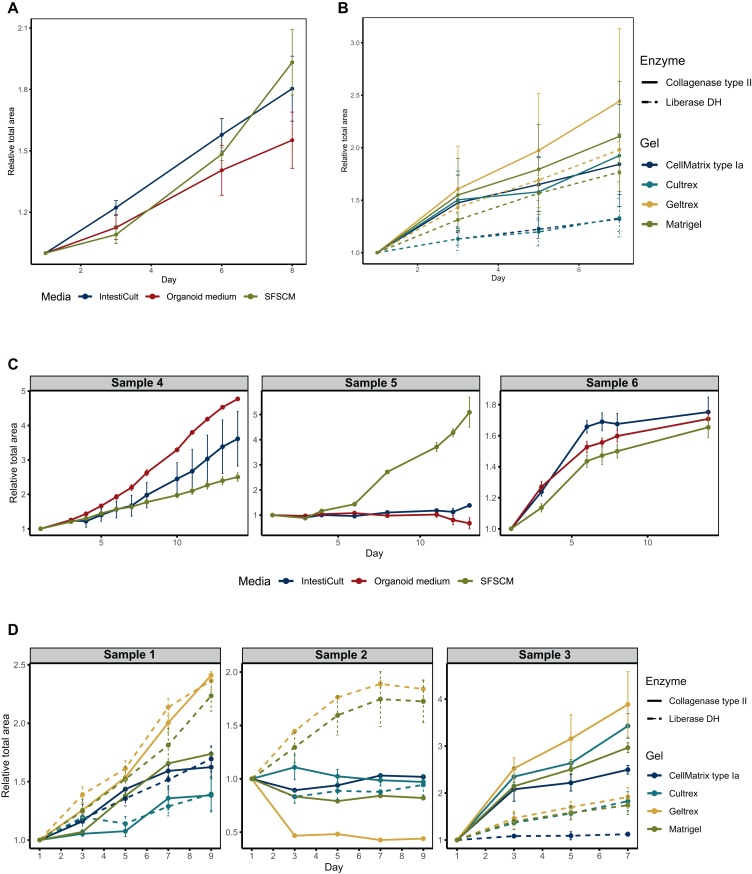
**Growth of patient-derived tumouroids under different cultivation methods and reagents.** (A) Continuous growth of samples 4-6 using either Organoid medium, IntestiCult, and SFSCM, analysed together across samples. Error bars show the standard error of the mean (SEM) of technical replicates. (B) Continuous growth of samples 1-3, analysed together across samples, per processing-cultivation route. Error bars show the SEM across all samples per processing-cultivation route. (C) Continuous growth of samples 4-6 using either Organoid medium, IntestiCult, or SFSCM, analysed individually per sample. Error bars show the SEM of four technical replicates. (D) Continuous growth of tumouroids from samples 1-3 per processing-cultivation route. Error bars show the SEM of two technical replicates. For sample 5, some wells had to be excluded from analysis due to gel breakage, resulting in the Liberase DH + CellMatrix type I-A condition being excluded. SFSCM, serum-free stem cell medium.

Tumouroid growth varied significantly across all samples. In combined analyses of samples 4-6, none of the growth media tested produced a clear growth difference (Figure 2A). Individual analysis (Figure 2C), however, showed that in sample 4, organoid-like growth media (IntestiCult™ and Organoid medium) promoted faster growth compared with the simpler SFSCM, whereas the opposite was true for sample 5. Sample 6 showed similar growth across all media, with a slight preference for the organoid-like ones.

For selection of digestion enzyme and ECM, combined analysis (Figure 2B) seems to show that digestion using collagenase type II and cultivation in Matrigel or Geltrex (both from the same source^31^) appeared to confer more rapid growth than digestion using Liberase DH with cultivation in Cultrex or Cellmatrix Type I-A. Also, however, here there were individual differences (Figure 2D). Sample 2 grew faster after being processed with Liberase DH and showed no discernible growth with collagenase type II. Sample 3, by contrast, displayed distinctly faster growth with collagenase type II. Sample 1 had mixed results with the enzymes but generally grew faster in Matrigel and Geltrex. This was also true for samples 2 and 3.

These results indicate substantial variation in sample growth across different digestion enzymes, ECMs, and growth media. Although our sample size was insufficient for statistically significant differences, we observed that some samples show better growth in certain media, while others show minimal differential sensitivity to reagent choice. We selected reagents that promoted faster growth in combined analyses or required less preparation, focusing on future scalability. We subsequently used this protocol to prepare samples 7-9.

### Image-based assessment reveals inter-sample heterogeneity in growth of colorectal tumouroids

Results from our initial experiments (Figure 2) indicated that increases in tumouroid size could be detected with non-invasive brightfield imaging. The number of tumouroids present in each image was limited, however, and many tumouroids were frequently out of focus in the brightfield images. We hypothesised that optimizing seeding density and applying more advanced imaging techniques using confocal microscopy could enhance both tumouroid growth and our accuracy in estimating it.

To test our hypothesis, we processed samples 7-9 according to the selected procedure (previous section) and seeded them at four different densities (250, 500, 1000, and 2000 tumouroids/well in a 24-well plate). An ImageXpress Micro Confocal High-Content Imaging System was used for imaging of tumouroids for 14 days following seeding.

All samples showed visible growth within the experimental timeframe (Supplementary Figures S2 and S3, Supplementary File B: Results, available at https://doi.org/10.1016/j.esmogo.2025.100137), and confocal imaging in combination with an optimised image analysis script enabled us to quantify this growth (Supplementary Figure S4, Supplementary File B: Results, available at https://doi.org/10.1016/j.esmogo.2025.100137). This held true across all seeding densities (Supplementary Figure S4, Supplementary File B: Results, available at https://doi.org/10.1016/j.esmogo.2025.100137). A seeding density of 1000 tumouroids per well generally showed the fastest growth and lowest growth variation (Supplementary Figures S4 and S5, Supplementary File B: Results, available at https://doi.org/10.1016/j.esmogo.2025.100137). This density allowed us to observe clear growth differences between samples (Supplementary Figure S2C, available at https://doi.org/10.1016/j.esmogo.2025.100137).

Our analysis demonstrates that optimising tumouroid seeding density enhances growth. Switching from brightfield to confocal microscopy simplified image acquisition and provided comparable results between conditions (Supplementary Figure S6, Supplementary File B: Results, available at https://doi.org/10.1016/j.esmogo.2025.100137). The analysis further shows that high-quality imaging can effectively be used to evaluate tumouroid growth and detect differences in unperturbed growth across samples.

### Different measures of tumouroid dimensions may be used for assessment of unperturbed tumouroid growth

To account for well-to-well variations in growth rate and to be able to put our samples on the same scale, we have used relative total area as a main readout for estimating sample growth. ImageJ does, however, provide additional readouts related to tumouroid dimensions (average area covered, size, diameter, and perimeter), shape (circularity), and count over time. These metrics were derived from the same images used to calculate relative total area. We next wanted to investigate if these readouts might yield complementary insights.

**Figure 3:**
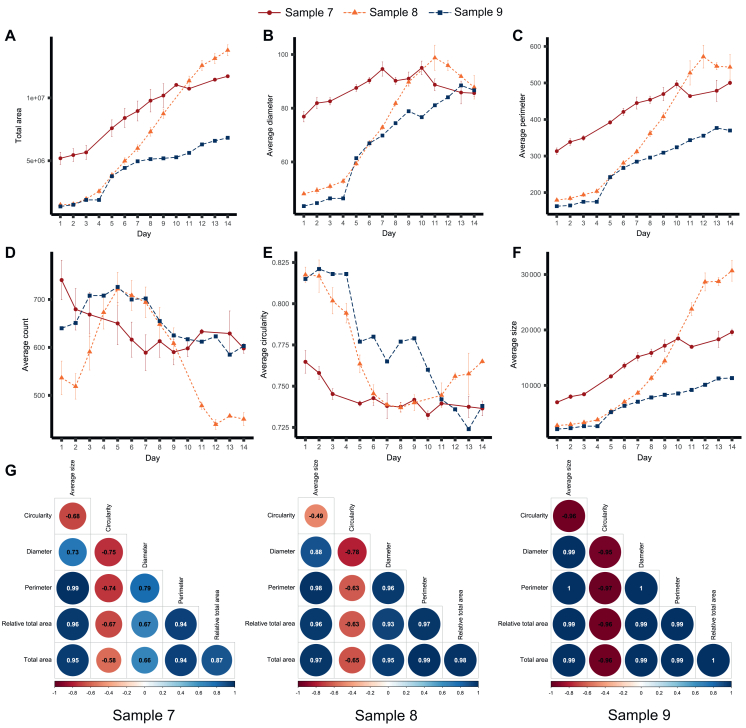
**Continuous analysis of additional imaging readouts of unperturbed wells from Samples 7-9.** (A) average total area (μm^2^), (B) average diameter (μm), (C) average perimeter (μm), (D) total count, (E) average circularity (ranges from 0 to 1), and (F) average size. Average diameter, perimeter, size, and circularity indicate the value for each of the readouts as the average across all analysed tumouroids per image, while total area is the average area covered by tumouroids in the whole well across technical replicates. Total count indicates the total number of tumouroids included in the analysis per image across technical replicates. Error bars show the standard error of the mean of four technical replicates. (G) Matrices show the Pearson correlation between all pairs of imaging readouts (except count) for samples 7 (left), 8 (middle), and 9 (right). Correlation coefficients are indicated by numbers in circles, relationship directionality by circle colour (blue = positive, red = negative), and correlation strength by circle size (larger circle = stronger correlation).

As with relative total area (Supplementary Figure S2B and C, available at https://doi.org/10.1016/j.esmogo.2025.100137), average tumouroid area, size, diameter, and perimeter displayed an upward trend over time (Figure 3A-C and F). This collective increase was consistent with visual observations and the geometrical relationships between these measures. Growth in tumouroid dimensions (area, size, diameter, perimeter) corresponded with a slight decrease in circularity for all samples (Figure 3E). Tumouroid count showed a rise-and-fall pattern for samples 8 and 9, whereas for sample 7, it steadily declined (Figure 3D).

Measures of average tumouroid size (area, diameter, perimeter) showed positive correlations with each other and negative correlations with circularity across all samples (Figure 3G). Stronger correlations were observed among readouts for samples 8 and 9 compared with sample 7. Additionally, relative total area and total area correlated strongly and positively with average area, diameter, and perimeter, and negatively with circularity, although slightly less so for sample 7. These results suggest that unperturbed tumouroid growth can generally be assessed using relative or average total area, with average tumouroid size, diameter, and perimeter serving as supplementary metrics in certain cases.

### Dose-dependent growth of perturbed tumouroids can be detected using different measures of tumouroid size

Next, we sought to investigate whether any of the previously studied measures could be used to assess the response of the tumouroids to drug perturbations. To study this, samples 7-9 were processed and seeded according to our selected protocol and treated with 0.1-1000 nM SN-38 in a five-step, 10-fold dilution series. Treatment was initiated 2 days after seeding and lasted for 7 days. The drug-containing medium was then removed and replaced with fresh growth medium. Imaging continued until the experiment had lasted a total of 14 days. Upon inspection of the images, a clear dose-dependent growth pattern was observed (Supplementary Figure S7, Supplementary File B: Results, available at https://doi.org/10.1016/j.esmogo.2025.100137).

**Figure 4:**
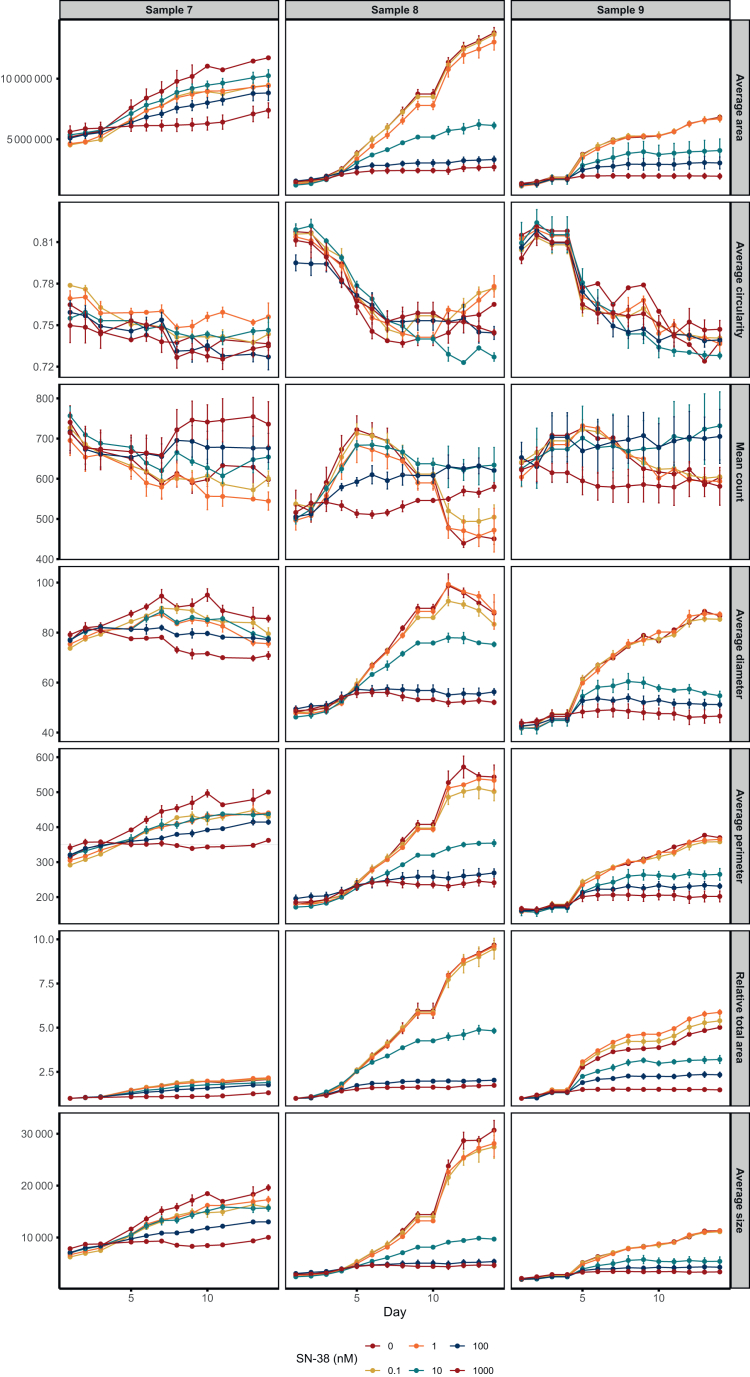
Monitoring of treatment response using continuous image-based readouts of average area (μm^2^), average circularity (0-1), total count, average diameter (μm), average perimeter (μm,) relative total area, and average size (μm^2^) for tumouroids of samples 7-9 upon treatment with 0.1-1000 nM SN-38. Average size, diameter, perimeter, and circularity indicate the value for each of the readouts as the average across all analysed tumouroids per image. Total count indicates the total number of tumouroids included in the analysis per image. Average total area and relative total area indicate the total area in each well covered by tumouroids. Different line colours indicate different concentrations of SN-38 (0.1, 1, 10, 100, and 1000 nM). Error bars show the standard error of the mean (SEM) of four technical replicates.

Analysis of time-course curves for all readouts (relative total area, average area, size, diameter, perimeter, count, and circularity) revealed a clear dose-dependent pattern in readouts of tumouroid dimensions (relative total area, average area, diameter, and perimeter), although dose-response relationships varied across samples. Dose-dependent responses were observed early in all size-related readouts, 3 days after initial drug exposure (experimental day 5), and became more pronounced over time. Circularity, unlike size-related readouts, steadily decreased throughout the experiment and showed greater fluctuation. This trend was observed across samples but was most notable in samples 8 and 9 (Figure 4). Tumouroid count, however, did not exhibit a clear dose-response relationship (Figure 4).

### Normalisation increases the relevance of response data from readouts estimating average tumouroid size

**Figure 5:**
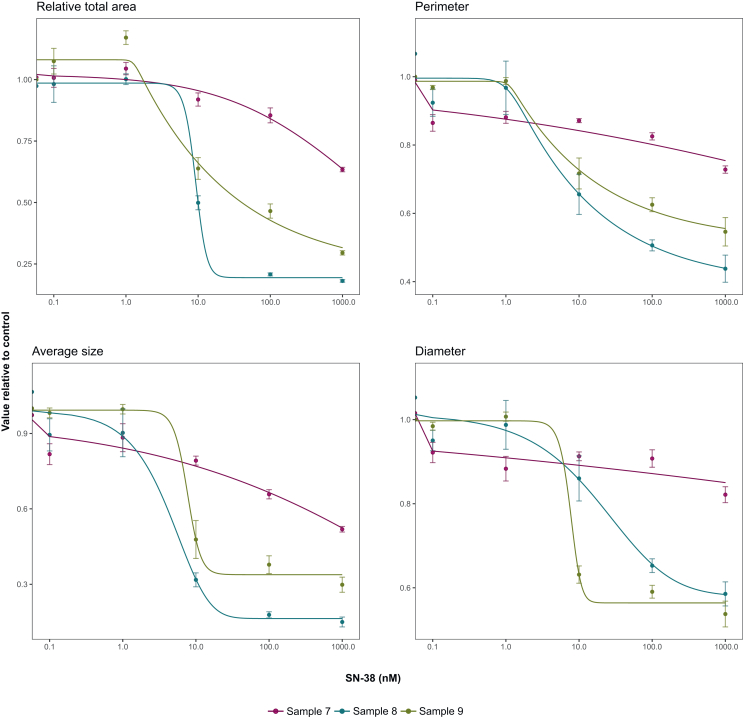
**Readout-dependent dose–response curves.** Dose–response curves per readout (relative total area, average perimeter, size, and diameter) for tumouroids of samples 7-9 upon treatment with 0.1-1000 nM SN-38. For each readout and treatment condition (sample, concentration), response is presented as the relative value compared with control (0.5% DMSO). Error bars show standard deviation of four technical replicates. The figure shows dose–response curves following 7 days of treatment with SN-38, and 5 additional days of culture without SN-38.

We concluded that measures related to tumouroid dimensions (total area, size, diameter, and perimeter) displayed the most pronounced growth differences between dosage levels. We next examined whether these readouts could generate dose–response curves to provide further insights into tumouroid sensitivity to SN-38. We used the previously collected response data, and calculated values relative to the vehicle control per dose. We used these values to fit dose–response models at various experiment days (Figure 5; Supplementary Figure S8, Supplementary File B: Results, available at https://doi.org/10.1016/j.esmogo.2025.100137).

Dose–response curves from models fitted to early experimental data (days 2, 6, 8, and 10; Supplementary Figure S8, Supplementary File B: Results, available at https://doi.org/10.1016/j.esmogo.2025.100137) indicate that robust dose–response relationships are evident as early as on day 6 of the experiment, i.e. 4-days post-drug exposure. The dose–response relationship appears to strengthen over time, along with the maximum observed growth inhibition relative to control wells.

Dose–response curves from day 14 (5 days post-drug removal; Figure 5) show varying shapes, indicating differences in sample sensitivity to SN-38. Although parameterisation of normalised dose–response curves varied by readout, they consistently identified sample 8 as most sensitive, followed by samples 9 and 7 (Figure 5), with one exception. The average diameter showed sample 9 as most sensitive, which is supported by predicted concentrations needed for achieving 50% growth inhibition relative to control (Supplementary Table S9, Supplementary File B: Results, available at https://doi.org/10.1016/j.esmogo.2025.100137). Predicted ED50 (predicted concentration needed for 50% of maximum observed growth inhibition; Supplementary Table S10, Supplementary File B: Results, available at https://doi.org/10.1016/j.esmogo.2025.100137) suggests sample 9 to be more sensitive than sample 8, even though it does not reach the same maximal inhibition.

In summary, these results demonstrate that several image-based readout parameters from our analysis method can be normalised to create dose–response models. These models reveal inter-sample variability in response to SN-38.

## Discussion

PDTs have emerged as a promising *in vitro* model for predicting individual patient responses to cancer treatment. Several studies demonstrate that tumouroid responses to clinically relevant therapies correspond with colorectal cancer patient responses retrospectively,^1^, ^2^, ^3^, ^4^, ^5^, ^6^, ^7^^,^^9^^,^^11^^,^^13^^,^^15^, ^16^, ^17^, ^18^, ^19^, ^20^, ^21^^,^^30^ and a few have prospectively guided patient treatment based on tumouroid responses,^8^^,^^10^^,^^12^^,^^14^^,^^22^ though with mixed success. These findings underscore the potential of this emerging model in personalised medicine, paving the way for what has come to be known as functional personalised medicine.^32^^,^^33^

Although the PDT model is clinically relevant, its widespread application in large-scale drug screening remains limited. We attribute this to the time-intensive nature of many tumouroid protocols, as well as limited reporting on method development. Most research groups opt to digest the material into a single-cell suspension to generate tumouroids (Supplementary Table S1, Supplementary File A: Materials and Methods, available at https://doi.org/10.1016/j.esmogo.2025.100137), a process that is not only time-consuming but also introduces risks of cell type and clonal selection. In this paper, we focus on optimising our protocol to perturb samples as close to their original state as possible, seeding them as clumps of cells rather than single cells, enabling us to generate dose–response data within a clinically relevant timeframe (14 days after biopsy). We report on several protocol choices made along the way, and our data suggest considerable tumour-to-tumour variation in how different reagents affect sample growth, which implies that we cannot know in advance the optimal reagents for each sample. We have chosen to focus on those experimental parameters that promoted the fastest growth in combined analyses or were least labour-intensive to prepare.

Given the typically small quantities of material available, PDT screening should ideally employ strategies that maximise data collection with minimally invasive and non-destructive techniques, like the image analysis method described here. Our data further suggest that monitoring sample growth continuously during drug exposure is essential, as dose–response can emerge early and vary in timing across samples and readouts. Many PDT studies, however, employ screening procedures with destructive, fixed-time endpoint readouts, such as CellTiter-Glo, to assess tumouroid response to treatment (Supplementary Table S1, Supplementary File A: Materials and Methods, available at https://doi.org/10.1016/j.esmogo.2025.100137). While these readouts can provide robust response measures at a single timepoint, they prevent continuous assessment and retrieval of viable culture material after treatment, as they typically involve cell fixation.

This study aimed to assess the suitability of label-free light microscopy brightfield or confocal imaging for collecting extensive continuous data on PDTs. To our knowledge, few studies have used imaging to assess tumouroid growth and response, and none with the same level of detail as in the present study. In previous studies, Yao et al.^21^ used image-based tumouroid area to quantify continuous drug response, while Pasch et al.^14^ assessed treatment response by measuring change in individual tumouroid diameter from start to end of treatment. Here, we continuously assessed tumouroid growth and response using area and diameter, together with several other image-based measures. Some of these measures were used to generate dose–response models that predicted clinically relevant drug sensitivities. Although none of the patients in this study’s drug–response experiments have received chemotherapy, generating dose–response models will help establish a classifier for drug sensitivity in future research.

This study used relative total area as a whole-well readout to assess growth for each well and timepoint. Normalising each well against itself was crucial for reducing variability among technical replicates, minimising the effect of well-to-well variation in seeding and growth rate. This is perhaps the most important advantage of our image-based readout, as it is impossible to carry out comparable intra-well normalisation using experimental strategies relying on destructive endpoint measurements. Our approach also placed samples on a common growth scale, despite differences in absolute total area, making comparison of samples easier. The data were also to fit dose–response models, allowing better evaluation of drug sensitivity. An important question for future research will be to determine which of our image-based readouts best align with patient responses.

One limitation of our image threshold method is a tendency towards overestimation of tumouroid-covered area when calculating relative total area: a global threshold method for image binarisation was utilised with an image-specific constant cut-off value for pixel classification. The cut-off value was selected on an individual image basis, based on pixel intensity distribution. As a consequence, more bright pixels could be positively classified at the experiment’s start than later, when larger and more opaque tumouroids appeared (Supplementary Figure S7, Supplementary File B: Results, available at https://doi.org/10.1016/j.esmogo.2025.100137).

We assessed average area, tumouroid size, diameter, perimeter, circularity, and count. While relative total area proved to be the most robust growth assessment across all samples, other parameters offered complementary insights. Additionally, metrics related to tumouroid dimensions (average area, diameter, perimeter) correlated strongly with each other and with relative total area, further supporting their use for growth assessment. In contrast to most other metrics, circularity showed a clear tendency to decrease over time. The same was true for count, although the trend was less pronounced compared with circularity. This is likely due to tumouroids overlapping as they grow, creating irregular shapes that lower the average circularity. In addition, these overlapping tumouroids may be classified as one instead of several, contributing to uncertainty regarding the number of tumouroids in image analyses.

While measures related to tumouroid dimensions were able to detect dose-dependent growth upon exposure to SN-38, one should keep in mind that some treatments induce tumouroid swelling before disruption and cell death.^34^ In such cases, using tumouroid dimensions as a growth measure could mislead interpretations, as swelling (indicating cell death) might be mistaken for growth. To avoid misinterpretation, image analysis should be routinely accompanied by manual inspection of images.

Our method requires adaptation to individual sample characteristics, which involves manual labour and inspection of images. We are currently working on automating the cultivation and image acquisition using robotics, to ease the manual workload. Regarding our image analysis method, some sample morphologies complicate the process, particularly due to overlapping tumouroids at experiment start (likely from high seeding density) or excessive single cells/cellular debris that obscure visible tumouroid growth. We believe that incorporating machine learning into our image analysis process could mitigate several of these challenges by minimising human discretionary assessments. It would also make tracking of individual tumouroids more feasible, which would allow evaluation of heterogeneous responses within the same well. Our image-based method is also sensitive to bubbles in media and/or ECM, infections, and gel breakage, with the latter two causing loss of technical replicates (although this is also a limitation in established, destructive readout methods). Finally, our data currently lacks a clinical correlate, as the patients from whom we derived drug-perturbed samples did not receive chemotherapy. This limits our ability to determine which image-based readouts best align with patient responses.

## Conclusion

Our study demonstrates that cultivation methods and reagents influence colorectal cancer tumouroid growth. We show that brightfield and confocal imaging with various size-related readouts are powerful tools for continuous, non-invasive monitoring of PDT growth, enabling detection of inter-sample variability in drug sensitivity—a promising avenue for clinical applications. We find relative total area to be the most robust readout for tumouroid growth assessment, but emphasise the value of integrating multiple readouts to enhance result reliability.

## Declaration of generative AI and AI-assisted technologies in the writing process

During the preparation of this work the author(s) used Chat-GPT 4.0 in order to improve readability and language. After using this tool/service, the author(s) reviewed and edited the content as needed and take(s) full responsibility for the content of the publication.
