# Corrigendum to “Continuous Imaging to Evaluate Growth and Drug Responses of Patient-Derived Colorectal Tumouroids”: ESMO Gastrointestinal oncology volume 7 (2025) 100137

**DOI:** 10.1016/j.esmogo.2025.100192

**Published:** 2025-06-20

**Authors:** B.C. Sakshaug, E. Folkesson, T.H. Haukaas, M.S. Sigfúsdóttir, H.H. Trøen, S.B. Sperstad, H.C.H. Bwanika, C. Ringers, I.A. Bergstrøm, G. Klinkenberg, T. Visnes, Å. Flobak

**Affiliations:** 1 Department of Clinical and Molecular Medicine, Norwegian University of Science and Technology, Trondheim, Norway; 2 Department of Biotechnology and Nanomedicine, SINTEF Industry, Trondheim, Norway; 3 The Cancer Clinic, St Olav’s University Hospital, Trondheim, Norway

The authors would like to inform of a minor corrigendum to our paper “Continuous Imaging to Evaluate Growth and Drug Responses of Patient-Derived Colorectal Tumouroids”, published in ESMO Gastrointestinal oncology March 2025. The corrigendum concerns Figure 5 and Supplementary Figure S8, which we have updated after revision of our code. The corrigendum does not lead to any major changes in the interpretation of our data, which is that confocal imaging and image-processing software can be utilized to capture dose-dependent growth in CRC-tumoroids upon perturbation. The paragraph following Figure 5 has also been revised.

Orignally published paragraph:

Dose–response curves from day 14 (5 days post-drug removal; Figure 5) show varying shapes, indicating differences in sample sensitivity to SN-38. Although parameterisation of normalised dose–response curves varied by readout, they consistently identified sample 8 as most sensitive, followed by samples 9 and 7 (Figure 5), with one exception. The average diameter showed sample 9 as most sensitive, which is supported by predicted concentrations needed for achieving 50% growth inhibition relative to control (Supplementary Table S9, Supplementary File B: Results, available at https://doi.org/10.1016/j.esmogo.2025.100137). Predicted ED50 (predicted concentration needed for 50% of maximum observed growth inhibition; Supplementary Table S10, Supplementary File B: Results, available at https://doi.org/10.1016/j.esmogo.2025.100137) suggests sample 9 to be more sensitive than sample 8, even though it does not reach the same maximal inhibition.

Revised paragraph:

Dose–response curves from day 14 (5 days post-drug removal; Figure 5) show varying shapes, indicating differences in sample sensitivity to SN-38. Although parameterisation of normalised dose–response curves varied by readout, they consistently seemed to identify sample 8 as most sensitive, followed by samples 9 and 7 (Figure 5), with the exception of average diameter, which seemed to identify sample 9 as most sensitive. Predicted concentrations needed for achieving 50% growth inhibition relative to control identified sample 8 as most sensitive, although this level of inhibition was not reached for several sample and readout combinations (Supplementary Table S9, Supplementary File B: Results, available at https://doi.org/10.1016/j.esmogo.2025.100137). Predicted ED50 (predicted concentration needed for 50% of maximum observed growth inhibition; Supplementary Table S10, Supplementary File B: Results, available at https://doi.org/10.1016/j.esmogo.2025.100137) suggests sample 9 to be more sensitive than sample 8, even though it does not reach the same maximal inhibition.

The authors would like to apologise for any inconvenience caused.

**Figure 5:**
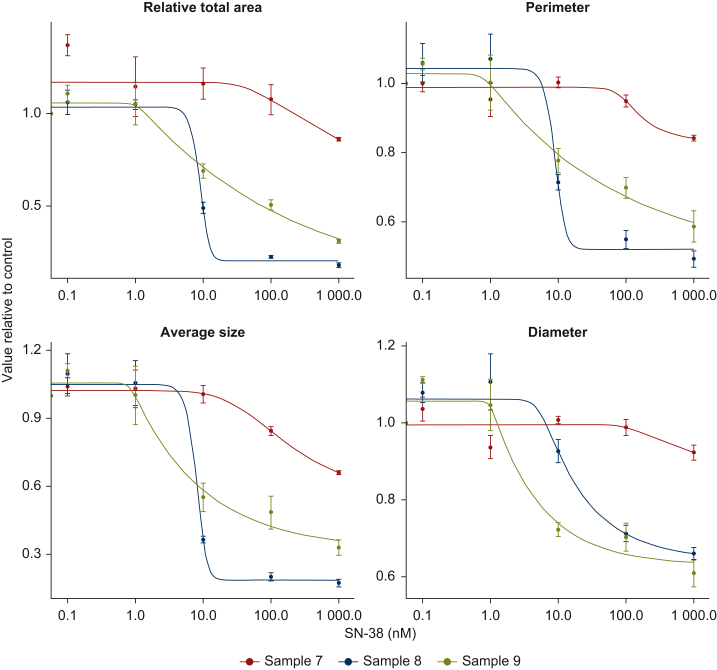
**Readout-dependent dose-response curves**. Dose-response curves per readout (relative total area, average perimeter, size, and diameter) for tumouroids of samples 7-9 upon treatment with 0.1-1000 nM SN-38. For each readout and treatment condition (sample, concentration) response is presented as the relative value compared to control (0.5% DMSO). Error bars show the standard error of the mean. The figure shows dose-response curves following 7 days of treatment with SN-38, and 5 additional days of culture without SN-38, for a total of 14 days in culture.

## Supplemental data

Supplementary Figure S8

R Script in ITEMS

