# Supplementary material for: Continuous imaging to evaluate growth and drug responses of patient-derived colorectal tumouroids
Source: ESMO Gastrointest Oncol. 2025 Mar 3;7:100137. doi: 10.1016/j.esmogo.2025.100137 (PMC12836542; doi:10.1016/j.esmogo.2025.100137)
Supplement: Supplemental Material [file mmc1.docx]

# Supplementary Material

Continuous Imaging to Evaluate Growth and Drug Responses of Patient-Derived Colorectal Tumouroids

Baard Cristoffer Sakshaug^1✢^, Evelina Folkesson^1,2✢^, Tonje Husby Haukaas^2✢^, Margrét Sylvía Sigfúsdóttir^2^, Hanne Hein Trøen^2^, Sigri Bakken Sperstad^2^, Henri Colyn Harry Bwanika^1^, Christa Ringers^1^, Ingrid Aune Bergstrøm^1^, Geir Klinkenberg^2^, Torkild Visnes^2^, Åsmund Flobak^1,2,3^

1. Department of Clinical and Molecular Medicine, Norwegian University of Science and Technology, Trondheim, Norway
2. Department of Biotechnology and Nanomedicine, SINTEF Industry, Trondheim, Norway
3. The Cancer Clinic, St Olav’s University Hospital, Trondheim, Norway

✢ These authors contributed equally: Evelina Folkesson, Baard Cristoffer Sakshaug, and Tonje Husby Haukaas

# Supplementary file A: Materials and methods

## Methods

### Protocol optimization

#### Selection of digestion enzyme and extracellular matrix

Following surgical removal of tumour tissue, a piece of the tissue was placed in 20 mL of DMEM supplemented with 100 U/mL penicillin-streptomycin (referred to as supplemented DMEM). The sample was washed twice in 20 mL of HBSS. Necrotic tissue was removed, and the remaining tissue was divided into two smaller pieces (Fig. M1) which were transferred to two separate 50-mL tubes filled with 20 mL of HBSS. Hereafter, the two pieces were handled separately. Using scalpels, each piece was minced into 1-2 mm tissue fragments which were transferred to a centrifuge tube and centrifuged at 200G for 5 minutes at 4°C. The procedure was followed by two washes (addition of 20 mL of HBSS, inversion of tube, centrifugation (200G, 5 minutes, 4°C) and removal of HBSS). The minced tissue was resuspended in 20 mL supplemented DMEM with digestion enzyme (1 mg/mL for Collagenase type II, 0.26 U/mL for Liberase DH) (Fig. M1) in a 100-mL conical flask with a magnet bar.

Digestion took place in a 37 °C water bath with constant stirring (digestion time: 2h for Liberase DH, 20 min for Collagenase type II). Following digestion, the digestate was transferred to a 50-mL centrifuge tube, centrifuged at 200G (5 minutes, 4°C), followed by medium removal. The sample was resuspended in 20 mL HBSS and sequentially filtered using 500 μm and 40 μm filters. Tumouroids collected in the 40 μm filter were transferred to a 50-mL centrifuge tube using a 1 mL pipette. The tube was centrifuged (200G, 5 minutes, 4°C), HBSS was discarded and the tumouroids underwent another round of washing (addition of 20 mL HBSS, inversion of tubes, centrifugation and removal of supernatant), whereafter they were resuspended in 0.4-1 mL of HBSS. The tumouroid suspension was aliquoted in four Eppendorf tubes, followed by centrifugation (200G, 5 minutes, 4°C) and removal of HBSS. Tumouroids were then resuspended in serum-free stem cell medium (SFSCM) and extracellular matrix (one type per Eppendorf tube) according to volumes given in Table M3. 50 μL gel drops were plated according to the layout in Figure M1; 2 wells per condition (enzyme and matrix type). Following 30 minutes of incubation at 37°C, the gel drops were overlaid with 500 μL SFSCM. Tumouroids were incubated overnight, whereafter the medium was changed. After this, the medium was changed every 2-3 days. The growth of the tumouroids was monitored by imaging using an EVOS FL Auto 2 imaging system for 14-21 days. Samples 1-3 were subjected to this process.

#### Selection of growth medium

Samples (4-6) were processed according to the protocol described above with the following exceptions: before mixing with Matrigel, samples were resuspended in supplemented DMEM (see above) instead of SFSCM. Additionally, the 40 μm filtrate was kept and processed in the same way as the 40 μm retentate, to see if the other growth media could support the growth of single cells. The samples were mixed 1:1 with Matrigel, and seeded in four technical replicates. After polymerization of the Matrigel, the samples were overlaid with 500 μl of either serum-free Stem Cell Medium (SFSCM), InstestiCult^TM^, or IBD-media (lab-made organoid growth medium, recipe can be seen in Supplementary Tables M4-6). Sample 6 is an exception to this, as only the 40 μm retentate was cultured, using SFSCM (8 technical replicates) and IntestiCult^TM^ (16 technical replicates.).

#### Selection of seeding density

Samples 7-9 were processed according to the optimized protocol (see above) with the following exception: following digestion, washing, and counting, four tumouroid suspensions with densities of 10, 20, 40 and 80 tumouroids/μL were prepared, rendering a final concentration of 250, 500, 1,000 and 2,000 tumouroids/drop of gel (50 µl) in plates. Each suspension was mixed 1:1 with Matrigel and seeded in six technical replicates. Medium was changed every 2-3 days. The growth of the tumouroids was monitored by imaging using an ImageXpress Micro Confocal High-Content Imaging System. Imaging was performed daily for 14 days.

#### Drug exposure

In addition to selection of seeding density, Samples 7-9 yielded enough material to allow for drug response evaluation. Samples were processed according to the selected protocol described in the main paper and seeded at a density of 500-1000 tumouroids per well in one 24-well plate, and cultivated for two days. After this, growth media was aspirated and replaced with drug-containing medium (SFSCM) with a 5-step 10-fold dilution of SN-38 ranging from 0.1-1,000 nM, as well as a DMSO-control. There were 4 technical replicates per condition. Samples were cultivated with drug-containing medium for 7 days, before it was removed and replaced with fresh growth medium. Samples were cultivated until the experiment had lasted for 14 days. Imaging was done continually throughout the experiment, usually every day to every other day.

### Scripts

#### Image analysis

Images were analysed using the script below. Before analysis, images were visually inspected to confirm growth, and subsequently subjected to a testing script which varied the “rolling” variable in the run(“Subtract Background…”) argument, as well as the “saturated” variable in the run(“Enhance Contrast…”) argument. The former took on the values 10, 50, and 100, while the latter took on the values 0.5, 1.5, 2.5, and 3.5. The values visually deemed to best fit the sample was then selected for processing of all the images. For some samples, the run(“Remove Outliers…”) argument had to be adjusted to remove larger objects due to a lot of debris in the images. Images from wells with bubbles in them were excluded only on the day they appeared, unless the bubble appeared on day 1, in which case the entire well was excluded from analysis, or the bubble was manually coloured black to be excluded from analysis if this was feasible without compromising results. Wells with broken gels were excluded from analysis from the first day they were observed to be broken in images. Wells with signs of infection, or other non-tumouroid debris, were excluded from analysis from the day the signs of infection or debris appeared.


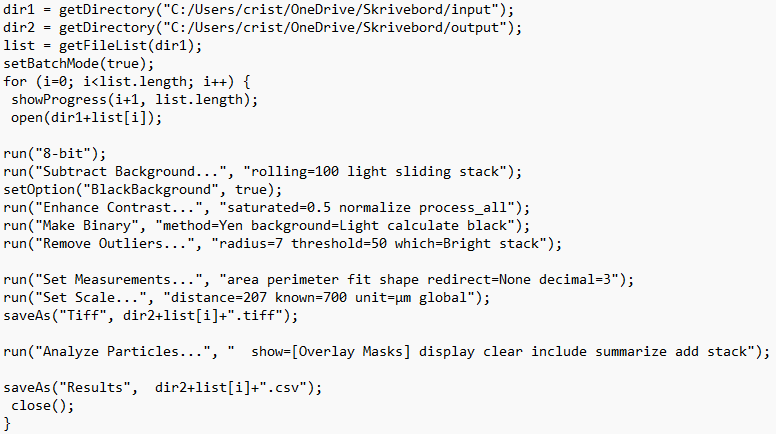


#### Data analysis

See separate R-notebook file.

## Figures and tables

**Table S1.** Protocol information from relevant papers cited in the main text.

| **Study** | **Enzyme** | **ECM** | **Medium** | **Seeding** | **Density** | **Readout** | **Time to result** | **Culture success rate** |
| --- | --- | --- | --- | --- | --- | --- | --- | --- |
| **Jeppesen et al.** | Collagenase type II | Matrigel | STEMPRO hESC SFM | Spheroids | 25 organoids/well in 96-well plates | Image-based | 2 weeks | 83% |
| **Vlachogiannis et al.** | TrypLe | Matrigel | Organoid-like | Single cells | 4500-6000 cells/well in 96-well plates | CellTiter-Blue | Not reported | 70% |
| **Kondo et al.** | Liberase DH and DNase I | Matrigel | STEMPRO hESC SFM | Spheroids | Not reported | ATPlite 1step kit | Not reported | Not reported |
| **Ganesh et al.** | Collagenase type XI, dispase type II, Tryple Express | Matrigel | Organoid-like | Single-cells | 20000-50000 cells/well in 24-well plates | CellTiter Glo | > 4 weeks. | 77% |
| **Ooft et al.** | Collagenase type XI and dispase type II | Matrigel | Organoid-like | Single cells | 1000 cells/well in 48-well plates | CellTiter-Glo | > 3 weeks | 63% |
| **Pasch et al.** | Collagenase and dispase | Matrigel | Organoid-like | Single cells | Not reported. | Image-based | Variable, but around 2 weeks | 76% |
| **Yao et al.** | Collagenase type II and IV, hyaluronidase, and dispase type II | Matrigel | Organoid-like | Single Cells | 200 organoids/well in 48-well plates | Image-based and CellTiter-Glo | Variable, but usually < 4 weeks | 77%–85.7% |
| **Janakiraman et al.** | Liberase DH | Cultrex | Organoid-like | Single cells | 1*10^6^ cells/2 mL of Cultrex, seeded in 40 μL droplets in a 6-well plate | CellTiter-Blue | Not reported | n.a.* |
| **Narasimhan et al.** | Collagenase type IV, dispase, hyaluronidase, and DNase type I | Matrigel | Organoid-like | Single cells | 800 cells/well in 384-well plates | CellTiter-Glo | Not reported | 68% |
| **Xu et al.** | Collagenase type IV, hyaluronidase, DNase type 1, and TrypLE Express | Matrigel or cellulosic sponges** | Organoid-like | Single cells | 15-20 000 organoids/mL in 96-well plates | CellTiter-Glo | Not reported | 100% |
| **Wang et al.** | Collagenase type II | Matrigel | STEMPRO hESC SFM | Single cells | 200-1000 organoids/mL in 384-well plates | CellTiter-Glo | 7-14 days | 70-80% |
| **Ooft et al.** | Collagenase type XI and dispase type II | Matrigel | Organoid-like | Single cells | 1000 cells/well in 48-well plates | CellTiter-Glo | > 3 weeks | 63% |
| **Park et al.** | Collagenase type II and dispase type II | Matrigel | Organoid-like | Single cells | 5000 cells/well in 96-well plates | CellTiter 96 AQUEOUS One solution | Approx. 4 weeks | 70% |
| **Cui et al.** | Collagenase A | Matrigel | Organoid-like | Single cells | 1000 cells/well in 96-well plates | CellTiter-Glo | 1 week | Not reported |
| **Cho et al.** | None | Matrigel | Organoid-like | Single cells | 1000-1500 cells/well in 96-well plates | CellTiter-Glo | Not reported | 75% |
| **Ding et al.** | Collagenase and DNase I | Micro-organosphere* | Organoid-like | Single cells | Approx. 3000 cells/well in 384-well plates | CellTiter-Glo | 2 weeks | 100% |
| **Hsu et al.** | Collagenase and dispase type II | Matrigel | Organoid-like | Single cells | 100-150 organoids/well, plate-size not reported | Image based | Not reported | Not reported |
| **Mo et al.** | Collagenase type II and IV, hyaluronidase, and dispase type II | Matrigel | Organoid-like | Single cells | 200 organoids/well In 48-well plates | CellTiter-Glo | Not reported | 80.6% |
| **Lv et al.** | Collagenase type II and IV, hyaluronidase, and dispase type II | Matrigel | Organoid-like | Single cells | 200 organoids/well in 48-well plates | Image-based | Not reported | 88% |
| **Tang et al.** | Collagenase type II | Matrigel | Not reported | Single cells | 200-1000 organoids/mL, plated in 384-well plates | CellTiter-Glo | Not reported | 78.3% |
| **Martini et al.** | Collagenase and DNase | Matrigel | Organoid-like | Single-cells | 500 cells/well in 96-well plates | MTS or CellTiter-Glo | Approx. 2 weeks | 60-80% |
| **Wang et al.** | Collagenase type II | Matrigel | Organoid-like | Single-cells | Not reported | CellTiter-Glo | Not reported | 79% |
| **Zhang et al.** | Collagenase type IV | Not used | OriCell® hESC Medium (Cyagen Biosciences) | Spheroids | Not reported | CellTiter-Lumi™ | 1 week | 92.3% |
| **Smabers et al.** | Collagenase type II and hyaluronidase | Matrigel | Organoid-like | Spheroids | 125 000 organoids/20 mL, 40 μL seeded in 384-well plates | CellTiter-Glo | Not reported | Not reported |

**Table S2.** List over materials and reagents used for preparation, cultivation, and treatment of patient-derived tumouroids.

| **Component** | **Reference number** | **Manufacturer** |
| --- | --- | --- |
| Amphotericin B | A2942 | Sigma Aldrich |
| bFGF | 13256-029 | Invitrogen |
| Cellmatrix Type I-A | 631-00651 | Nitta Gelatin Inc. |
| Collagenase type II | 17101015 | Gibco |
| Dulbecco's Modified Eagle Medium (DMEM) | D5671 | Sigma Aldrich |
| Fetal Bovine Serum (FBS) | F7524 | Sigma Aldrich |
| 500 μm filters | 43-50500-50 | pluriSelect |
| 200 μm filters | 43-50200-03 | pluriSelect |
| 100 μm filters | 431752 | Corning |
| 40 μm filters | 431750 | Corning |
| Hanks' Balanced Salt Solution (HBSS) | H9269 | Sigma Aldrich |
| Liberase DH | 5401054001 | Roche Diagnostics |
| Matrigel | 356231 | Corning |
| 2-mercaptoethanol | 21985023 | Gibco |
| Penicillin-Streptomycin | 15140-122 | Gibco |
| SN-38 | S4908 | Selleckchem |
| StemPro™ hESC SFM | A10006-01 | Gibco |
| 24-well flat bottom cultivation plates | 3524 | Corning |
| Y-27632 (ROCK inhibitor) | SCM075 | Sigma Aldrich |
| adDMEMF12 | 12634-010 | ThermoFischer |
| B27 | 12634-010 | ThermoFischer |
| GlutaMAX | 35050-061 | ThermoFischer |
| N-acetyl cysteine | A9165 | Sigma Aldrich |
| Nicotinamide | N0636 | Sigma Aldrich |
| hEGF | AF-100-15 | Peprotech |
| A83-01 | 2939 | Tocris Biosciences |
| Wnt3a | GF-160 | Millipore |
| Gastrin | G9145 | Sigma Aldrich |
| R-spondin | 120-38 | Peprotech |
| SB202190 | S7067 | Sigma Aldrich |
| Noggin | 120-10C | Peprotech |
| IntestiCult^TM^ Organoid Growth Medium (Human) | 06010 | Stemcell Technologies |

**Table S3.** Volumes used for preparation of extracellular matrices (protocol optimization).

| **Type** | **Volume gel  (μL)** | **Volume SFSCM  (μL)** | **Volume reconstitution buffer (μL)** |
| --- | --- | --- | --- |
| Matrigel | 50 | 50 | - |
| Cellmatrix Type I-A | 70 | 20 | 10 |
| Cultrex | 67 | 33 | - |
| Geltrex | 67 | 33 | - |

**Table S4.1** Reagents used for preparation of Minigut A.

|  | **Concentration** | **Amount** |
| --- | --- | --- |
| Wnt-3A conditioned medium |  | 500 mL |
| BSA | 1 % | 10 g |
| GlutaMAX 100X | 1 x | 10 ml |
| HEPES 1M | 10 mM | 10 mL |
| Penicillin-streptomycin (10 000U/mL) | 100 U/mL | 10 mL |
| N2 Supplement 100X | 1 x | 10 mL |
| B27 Supplement 50X | 10 x | 20 mL |
| **Table S4.2** Reagents used for preparation of Minigut B | | |
|  | **Concentration** | **Amount** |
| Advanced DMEM/ F12 |  | 1000 mL |
| BSA | 1 % | 10 g |
| GlutaMAX 100X | 1 x | 10 mL |
| HEPES 1M | 10 mM | 10 mL |
| Penicillin-streptomycin (10 000U/mL) | 100 U/mL | 10 mL |
| N2 Supplement 100x | 1 x | 10 mL |
| B27 Supplement 50x | 1 x | 20 mL |
| **Table S4.3** Reagents used for preparation of Minigut C, which is referred to as “organoid medium” in the main text. | | |
|  | **Concentration** | **Amount** |
| Minigut A | 50% | 100 mL |
| Minigut B | 50% | 60 mL |
| Nicotinamide stock (122.12mg/mL) | 1:100 | 2000 μL |
| N-Acetyl-Cysteine stock (163.19mg/mL) | 1:1000 | 200 μL |
| Noggin Protein Stock (0.1mg/mL) | 1:1000 | 200 μL |
| R-spondin stock (1mg/mL) | 20% | 40 mL |
| A-83-01 stock (0.21mg/mL) | 1:1000 | 200 μL |
| SB202190 stock (9.94 mg/mL) | 1:3000 | 66.67 μL |
| Human EGF stock (0.5mg/mL) | 1:10 000 | 20 μL |
| [Leu] 15-Gastrin 1 stock (0.21mg/mL) | 1:10 000 | 20 μL |
| ROCK-inhibitor stock (3.20mg/mL) | 1:1000 | 200 μL |

**Table S5** Reagents used for preparation of SFSCM

|  | **Concentration** | **Amount** |
| --- | --- | --- |
| DMEM/F12 + Glutamax-I | 1 X | 15.8925 mL |
| StemPro hESC Supplement | 1 X | 0.4 mL |
| BSA 25% | 1.8 % | 1.44 mL |
| FGFb (10μ/mL) | 8 ng/mL | 16 μL |
| 2-Mercaptoethanol (55mM) | 0.1 mM | 36.4 μL |
| Penicillin-streptomycin (10 000U/mL) | 100 U/mL | 0.2mL |
| Amphothericin B (250 μg/mL) | 25 μg/mL | 2 mL |
| ROCK-inhibitor (12.9 mM) | 10 μM | 15.5 μL |

**Table S6** Reagents used for preparation of IntestiCult^TM^. The two first components are both included when purchasing the IntestiCult^TM^ Organoid Growth Medium (Human) listed in Table M1, while remaining reagents are acquired separately.

|  | **Concentration** | **Amount** |
| --- | --- | --- |
| IntestiCult™ OGM Human Basal Medium | 1X | 8.895 mL |
| Organoid Supplement | 1X | 8.895 mL |
| Penicillin-streptomycin (10 000U/mL) | 100 U/mL | 200 μL |
| ROCK-inhibitor (12.9 mM) | 10 μM | 15 μL |
| Amphothericin B (250 μg/mL) | 25 μg/mL | 2 mL |

**Table S7.** List over sample ID and assigned procedures for samples included in the study.

| **Sample ID** | **Procedure** |
| --- | --- |
| Sample 1 | Optimization of protocol parameters (digestion enzyme, extracellular matrix) |
| Sample 2 |  |
| Sample 3 |  |
| Sample 4 |  |
| Sample 5 |  |
| Sample 6 |  |
| Sample 7 | Optimization of protocol parameters (seeding density);  Optimization of imaging |
| Sample 8 |  |
| Sample 9 |  |

**Table S8.** R packages used for data analysis and/or graphics

| **Package name** | **Version** |
| --- | --- |
| tidyverse | 1.3.2 |
| ggplot2 | 3.5.1 |
| ggpubr | 0.6.0 |
| dplyr | 1.0.10 |
| drc | 3.0.1 |
| ggcorrplot | 0.1.4.1 |
| corrplot | 0.92 |
| cowplot | 1.1.3 |
| viridis | 0.6.5 |
| svglite | 2.1.3 |


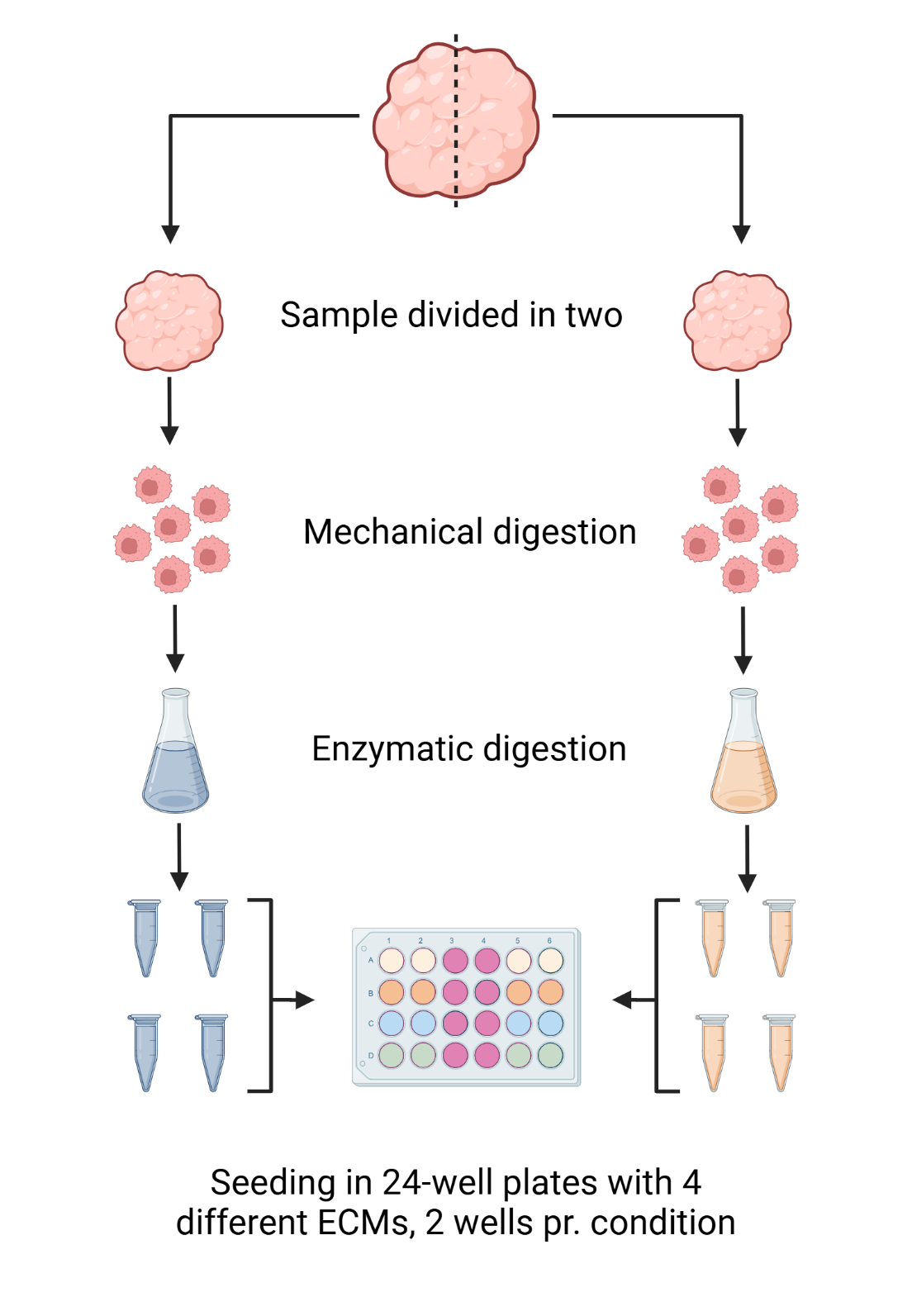


**Figure S1.** Simplified schematic overview of the procedure for testing of digestion enzyme and extracellular matrix. Created in BioRender. Sakshaug, C. (2024) https://BioRender.com/s52k852.

# Supplementary file B: Results

## Figures and tables

**
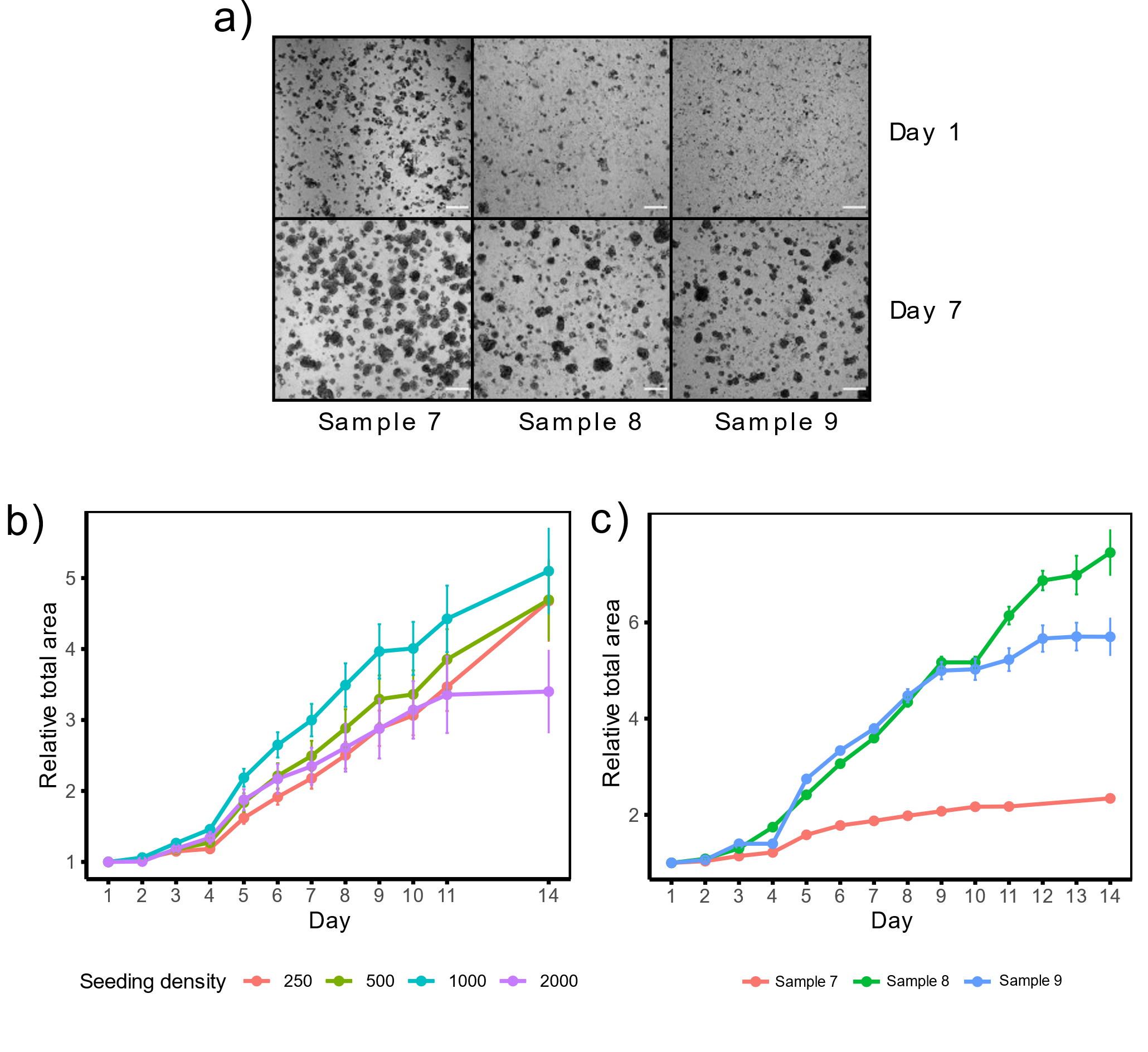
**

**Figure S2.** Image-based analysis of total tumouroid growth. **(a)** Brightfield images of Samples 7-9 on days 1 and 7 following seeding, under unperturbed conditions. Seeding density: 1,000 tumouroids/well. Scale bar = 500 µm. **(b)** Relative total area over time across Samples 7-9 seeded at 4 different densities, expressed as tumouroids/well. Error bars show the SEM of 12 technical replicates. **(c)** Relative total area over time for Samples 7-9 seeded at a 1000 tumouroids/well. Error bars show the SEM of 4 technical replicates.


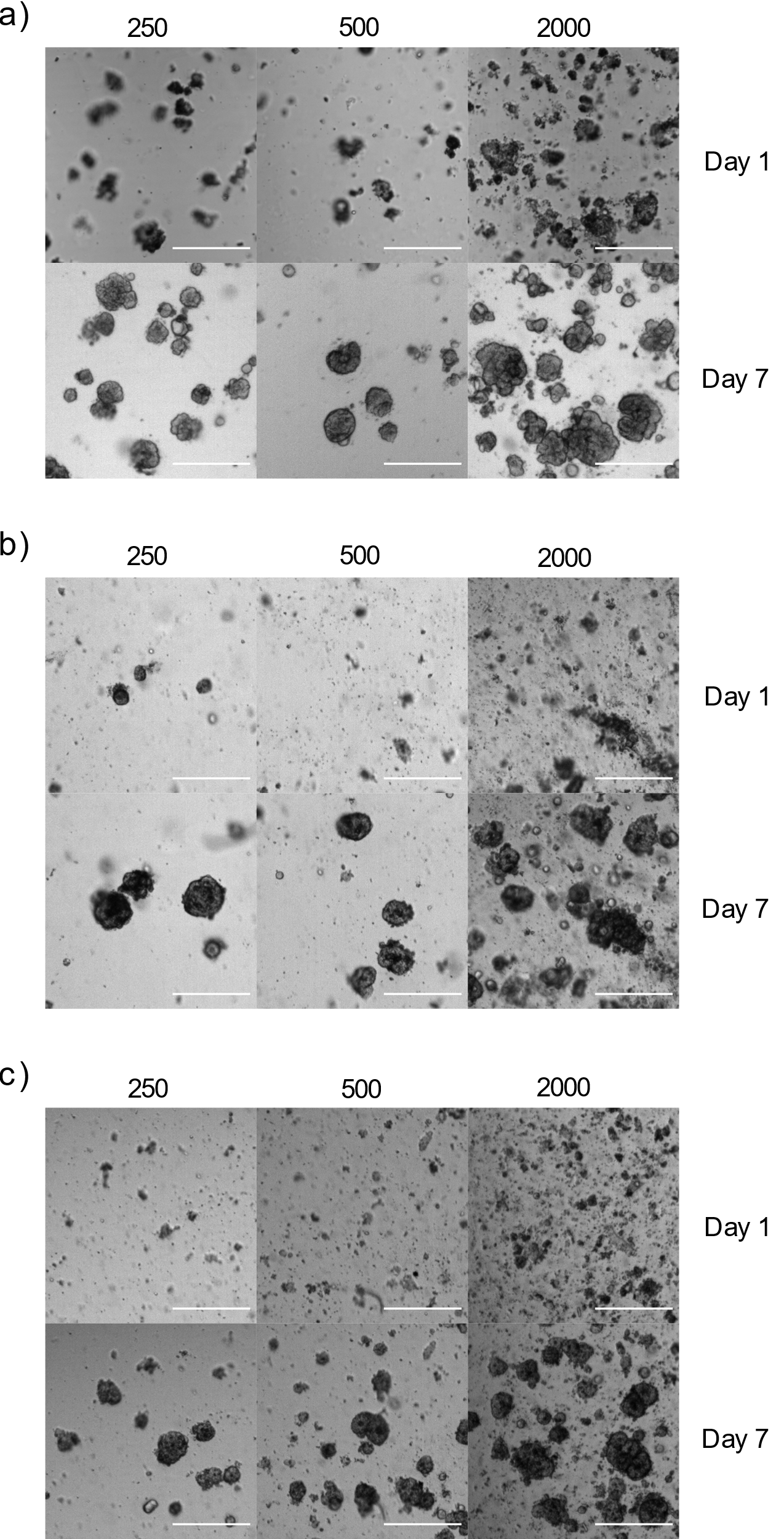


**Figure S3.** Brightfield images per sample and density. Brightfield images per density (250, 500, 2000 tumouroids/well) on day 1 and 7 for samples **(a)** 7, **(b)** 8, and **(c)** 9. Scale bar = 500 µm.

**
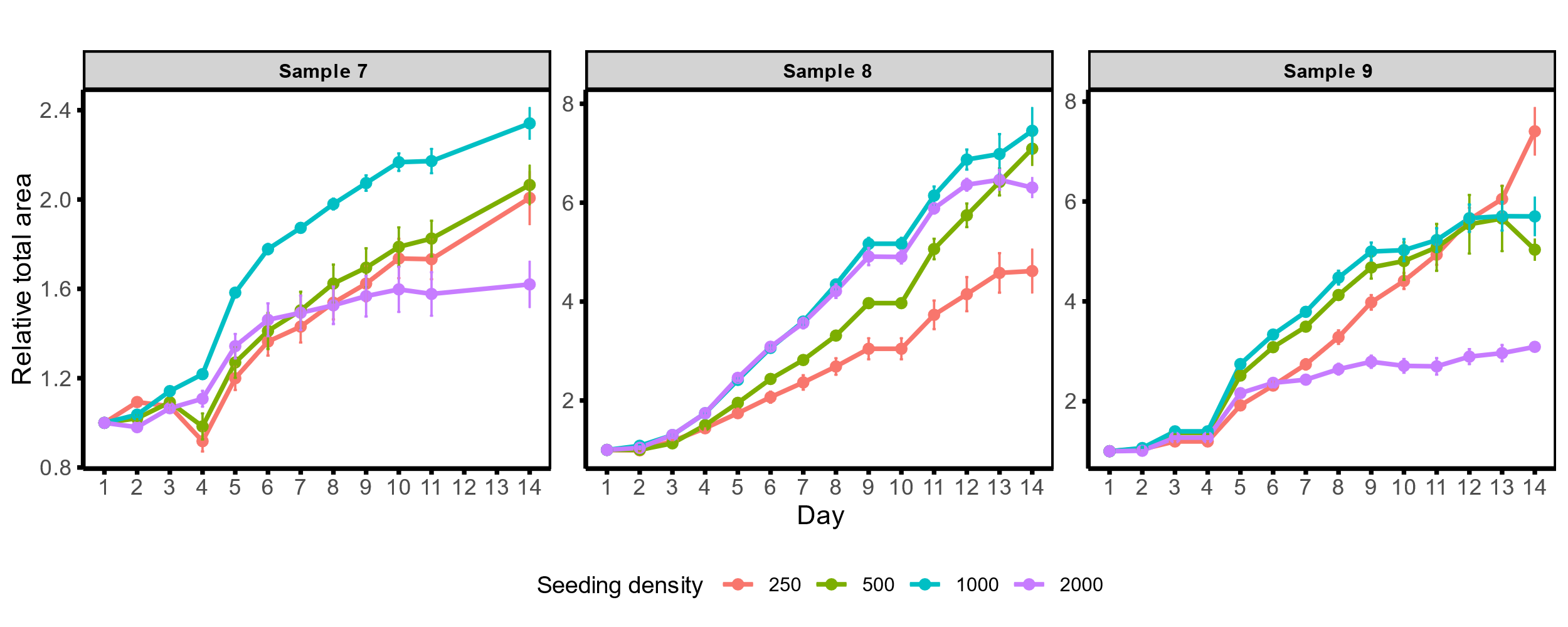
**

**Figure S4.** Growth of tumouroids seeded at four different densities (250, 500, 1000 and 2000 tumouroids/well). Growth curves show sample-and-density-specific growth for samples 7-9. Error bars show standard error of the mean of 4 technical replicates.


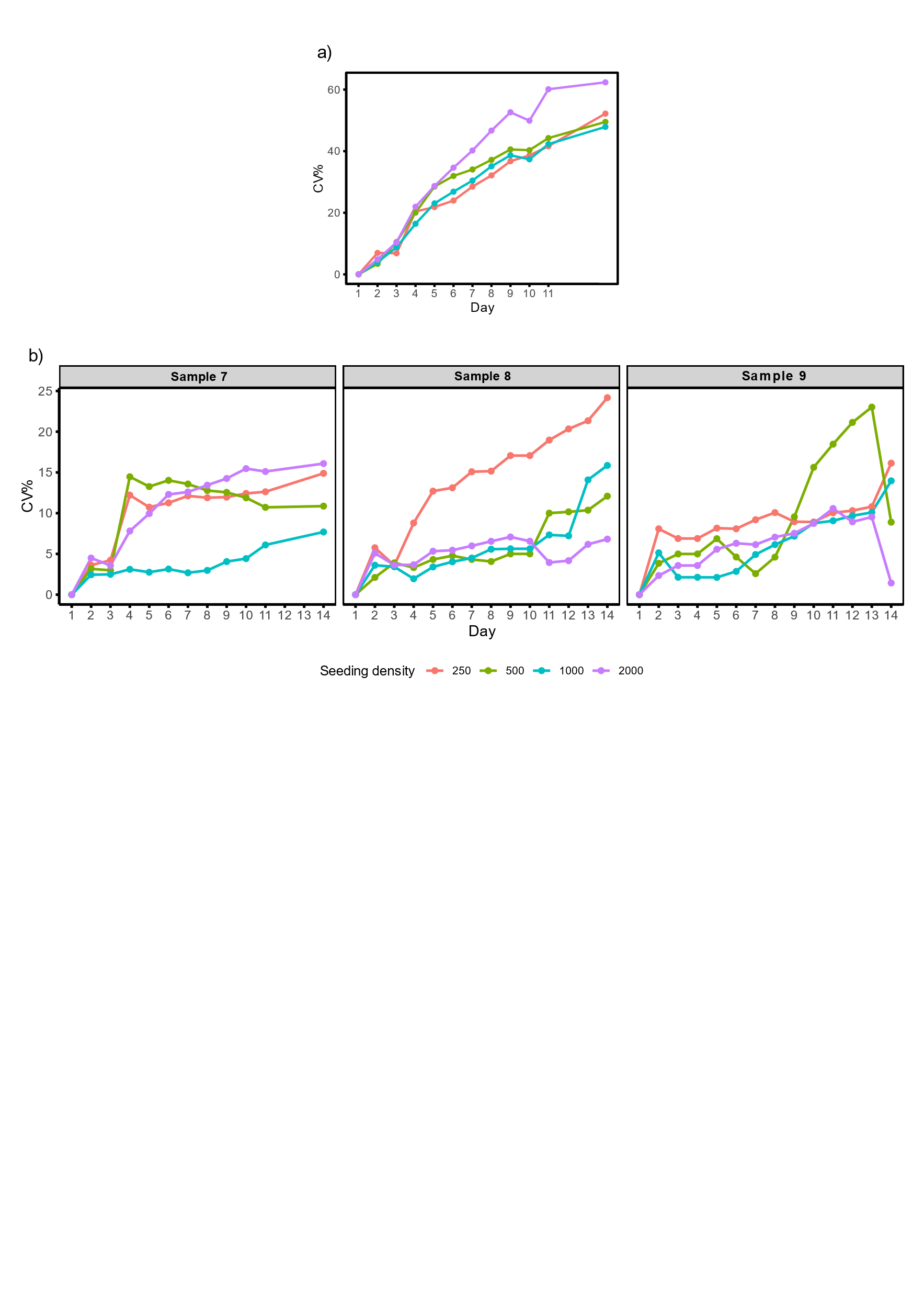


**Figure S5.** Coefficient of variation (CV) over time for tumouroids seeded at 250, 500, 1000 and 2000 tumouroids/well. **(a)** The curves show the average (CV) of three samples per density. **(b)** Sample-and-density-specific CV for samples 7-9.

**
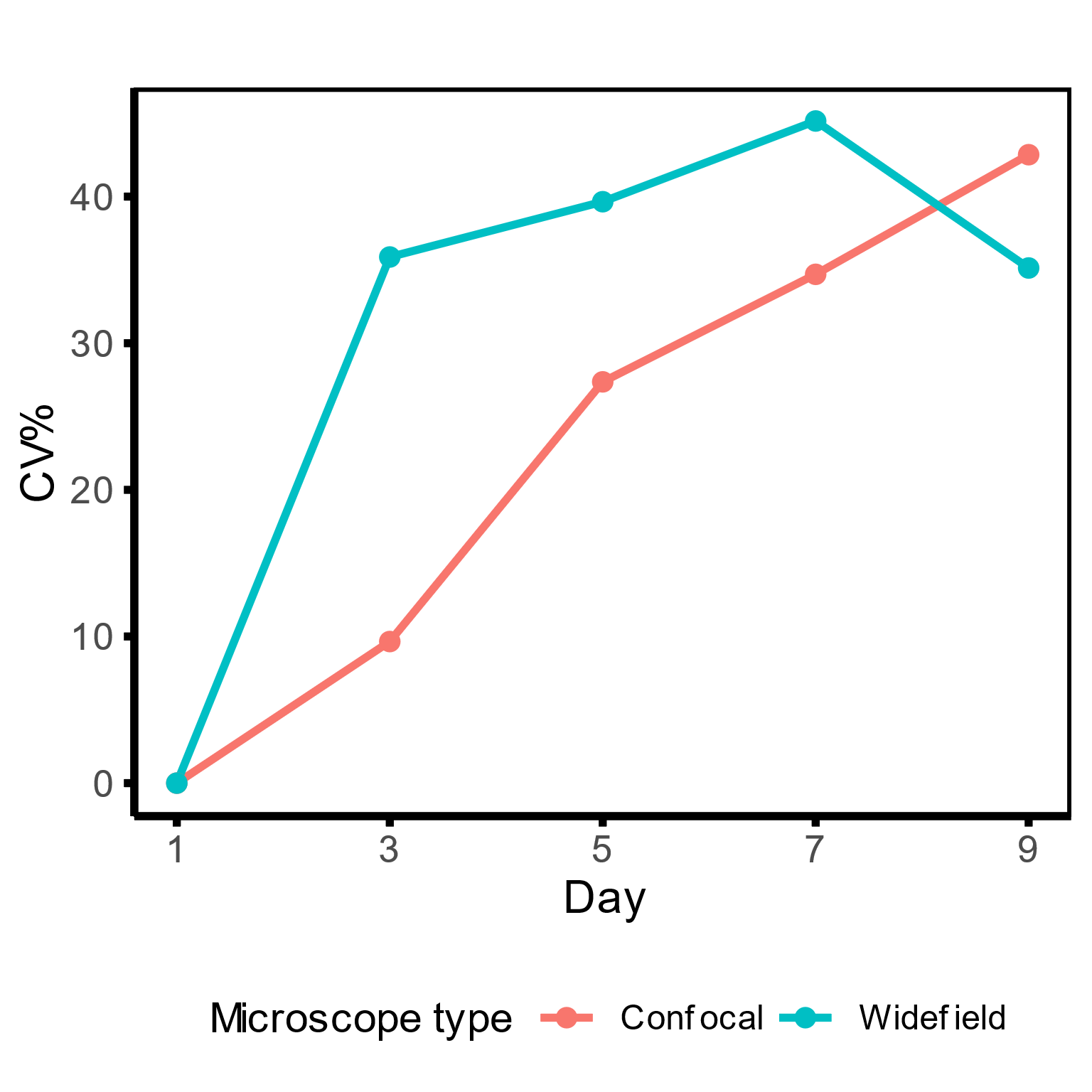
**

**Figure S6.** Coefficient of variation (CV) over time for tumouroids imaged with a widefield microscope (EVOS2, samples 1-3) and confocal microscope (ImageXpress, samples 7-9).


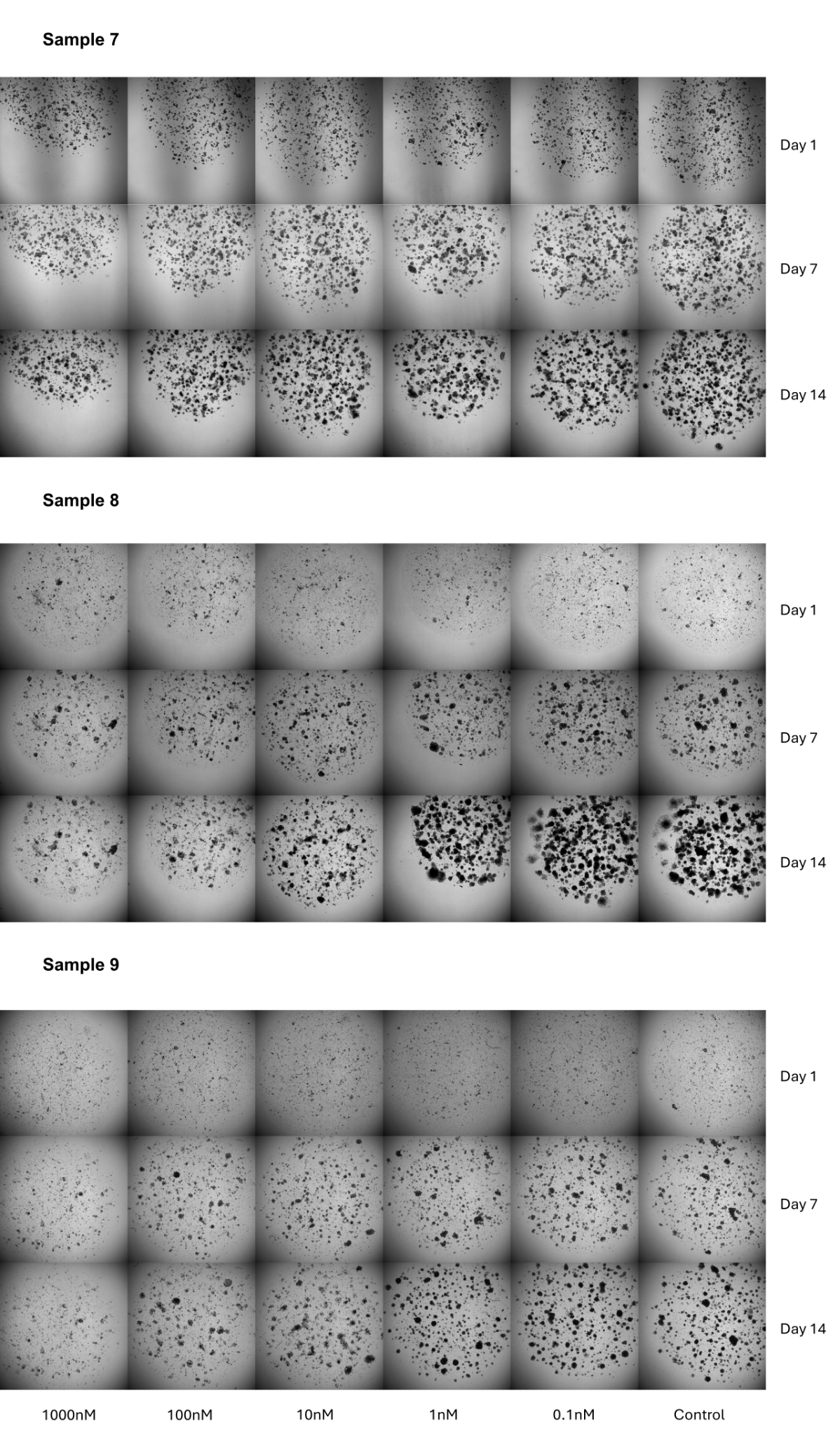


**Figure S7.** Brightfield images of Samples 7-9, captured on days 1, 7, and 14 of the drug exposure experiment.


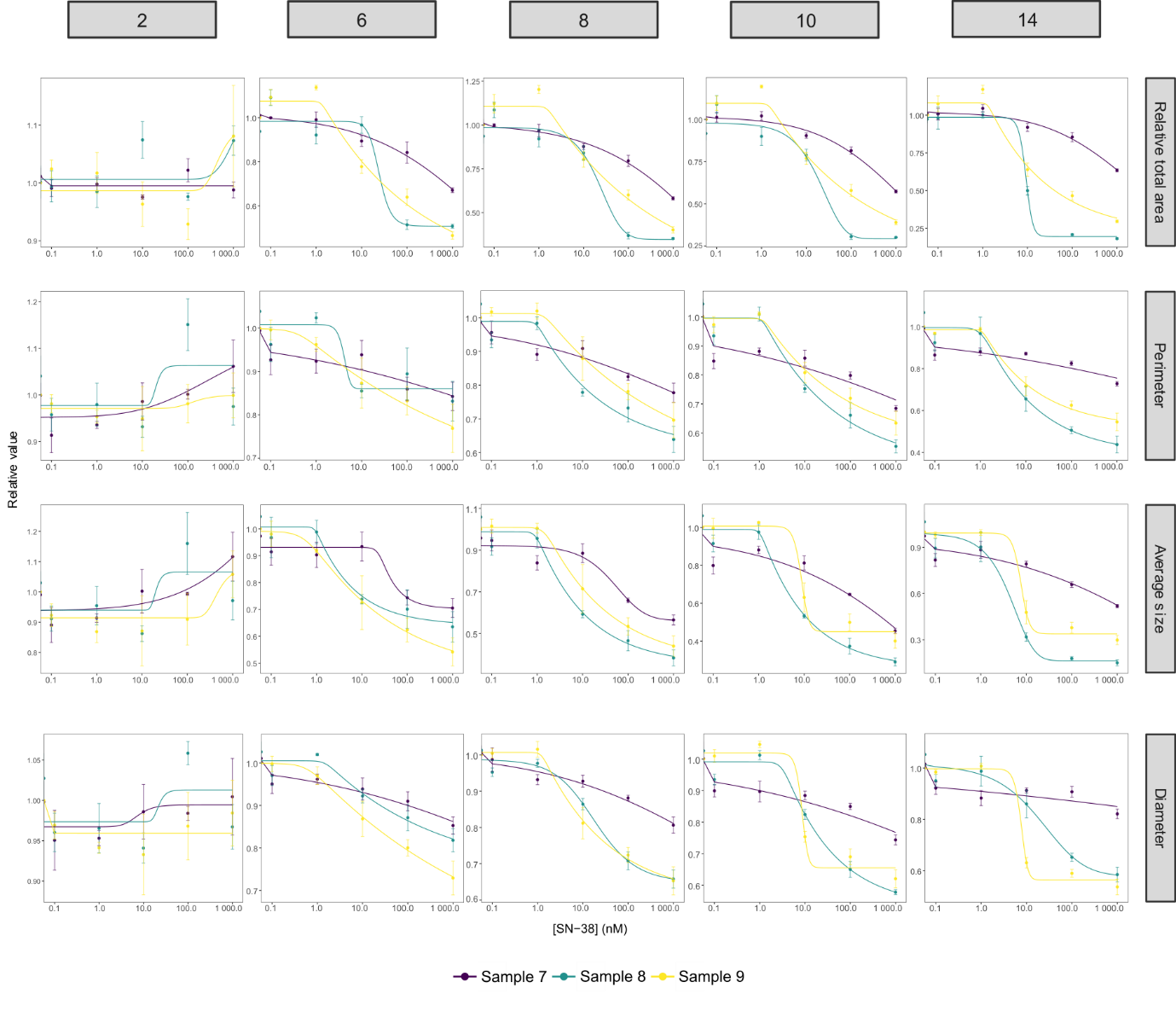


**Figure S8.** Day-and-readout-dependent dose-response curves. Dose-response curves per readout (relative total area, average size, diameter, and perimeter) and day (2, 6, 8, 10, and 14) for tumouroids of samples 7-9 upon treatment with 0.1-1000 nM SN-38. For each readout, day, and treatment condition (sample, concentration), response is presented as the relative value compared to control (0.5% DMSO). Error bars show standard error of the mean for 4 technical replicates.

**Table S9.** Predicted concentration needed to reach 50% growth inhibition compared to vehicle control for selected measures in samples 7-9 for SN-38 at day 14 of the experiment, or 5 days after removal of drug exposure. All concentrations are in nM. NA indicates that the generated 5-parameter log-logistic model did not reach 50% growth inhibition.

| **Measure** | **Sample** | | |
| --- | --- | --- | --- |
|  | **7** | **8** | **9** |
| **Relative total area** | 3453.60 | 9.99 | 40.22 |
| **Perimeter** | 13551726.00 | 110.77 | NA |
| **Average size** | 1401.70 | 5.58 | 9.62 |
| **Diameter** | 2.6410e+15 | NA | NA |

**Table S10.** Predicted concentration needed to reach 50% of maximal growth-inhibiting effect (ED50) for selected measures in samples 7-9 for SN-38 at day 14 of the experiment, or 5 days after removal of drug exposure. All concentrations are in nM.

| **Measure** | **Sample** | | |
| --- | --- | --- | --- |
|  | **7** | **8** | **9** |
| **Relative total area** | 125.30 | 9.83 | 1.27 |
| **Perimeter** | 37.5 | 1.08 | 1.09 |
| **Average size** | 179.8 | 30.74 | 8.04 |
| **Diameter** | 2.50 | 100.63 | 8.89 |


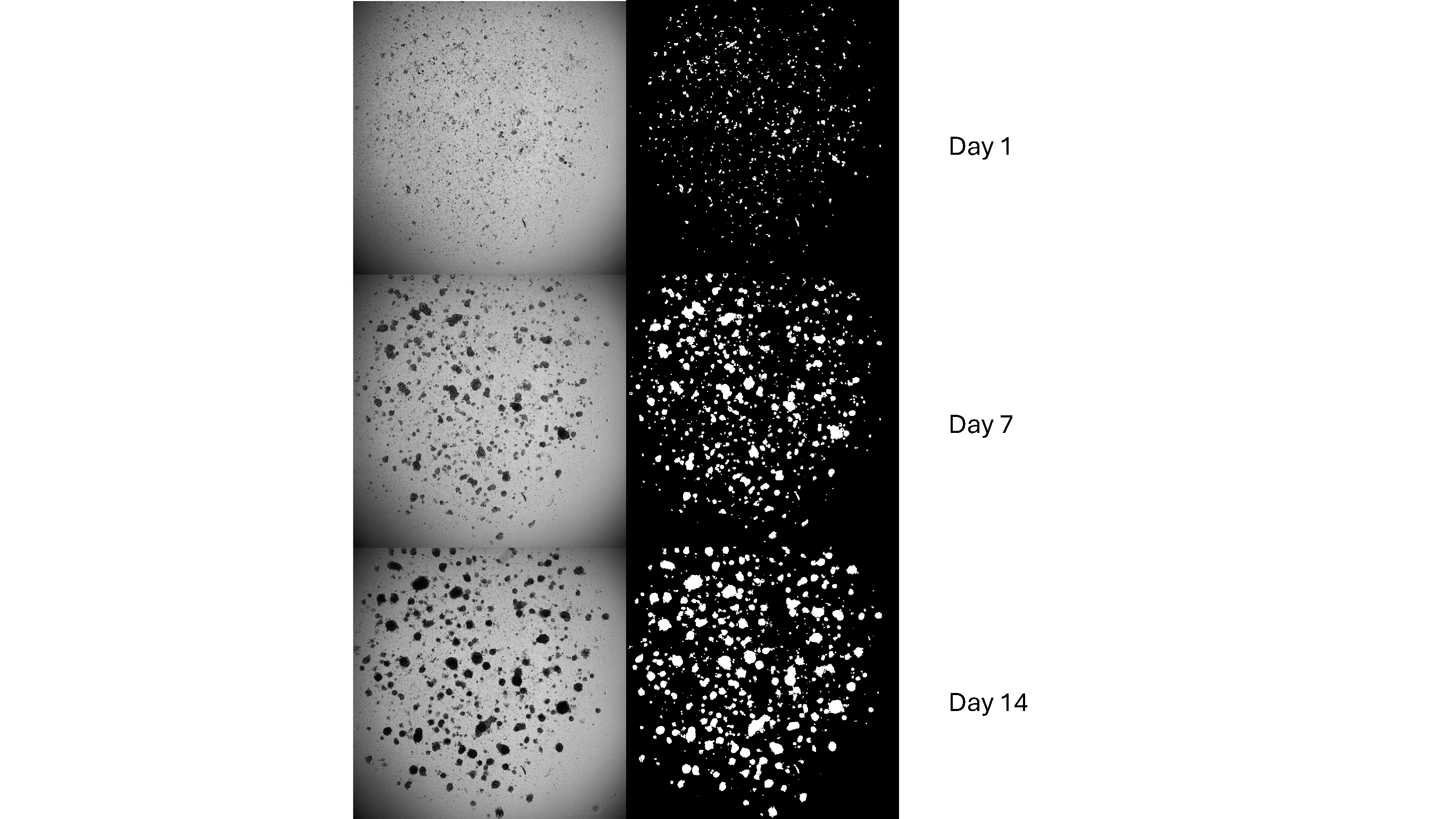


**Figure S9.** Brightfield and binarised images of Sample 9 tumouroids, captured on days 1, 7, and 14 of the drug exposure experiment. Images show tumouroids that have been exposed to 0.1 nM of SN-38. Binarized images have been created with the ImageJ-script shown in Supplementary File 1 – Materials and Methods.
